# Nationwide Surveillance of Influenza during the Pandemic (2009–10) and Post-Pandemic (2010–11) Periods in Taiwan

**DOI:** 10.1371/journal.pone.0036120

**Published:** 2012-04-24

**Authors:** Jen-Hsiang Chuang, Angela S. Huang, Wan-Ting Huang, Ming-Tsan Liu, Jih-Haw Chou, Feng-Yee Chang, Wen-Ta Chiu

**Affiliations:** 1 Epidemic Intelligence Center, Centers for Disease Control, Taipei, Taiwan, Republic of China; 2 Institute of Biomedical Informatics & Institute of Public Health, National Yang-Ming University, Taipei, Taiwan, Republic of China; 3 Field Epidemiology Training Program, Centers for Disease Control, Taipei, Taiwan, Republic of China; 4 Research and Diagnostic Center, Centers for Disease Control, Taipei, Taiwan, Republic of China; 5 Deputy Director-General's Office, Centers for Disease Control, Taipei, Taiwan, Republic of China; 6 Director-General's Office, Centers for Disease Control, Taipei, Taiwan, Republic of China; 7 Department of Internal Medicine, National Defense Medical Center, Taipei, Taiwan, Republic of China; 8 Minister's Office, Department of Health, Taipei, Taiwan, Republic of China; 9 School of Medicine, Taipei Medical University, Taipei, Taiwan, Republic of China; Centro de Biología Molecular Severo Ochoa (CSIC-UAM), Spain

## Abstract

**Introduction:**

Although WHO declared the world moving into the post-pandemic period on August 10, 2010, influenza A(H1N1) 2009 virus continued to circulate globally. Its impact was expected to continue during the 2010–11 influenza season. This study describes the nationwide surveillance findings of the pandemic and post-pandemic influenza periods in Taiwan and assesses the impact of influenza A(H1N1) 2009 during the post-pandemic period.

**Methods:**

The Influenza Laboratory Surveillance Network consisted of 12 contract laboratories for collecting and testing samples with acute respiratory tract infections. Surveillance of emergency room visits and outpatient department visits for influenza-like illness (ILI) were conducted using the Real-Time Outbreak and Disease Surveillance system and the National Health Insurance program data, respectively. Hospitalized cases with severe complications and deaths were reported to the National Notifiable Disease Surveillance System.

**Results:**

During the 2009–10 influenza season, pandemic A(H1N1) 2009 was the predominant circulating strain and caused 44 deaths. However, the 2010–11 influenza season began with A(H3N2) being the predominant circulating strain, changing to A(H1N1) 2009 in December 2010. Emergency room and outpatient department ILI surveillance displayed similar trends. By March 31, 2011, there were 1,751 cases of influenza with severe complications; 50.1% reported underlying diseases. Of the reported cases, 128 deaths were associated with influenza. Among these, 93 (72.6%) were influenza A(H1N1) 2009 and 30 (23.4%) A(H3N2). Compared to the pandemic period, during the immediate post-pandemic period, increased number of hospitalizations and deaths were observed, and the patients were consistently older.

**Conclusions:**

Reemergence of influenza A(H1N1) 2009 during the 2010–11 influenza season had an intense activity with age distribution shift. To further mitigate the impact of future influenza epidemics, Taiwan must continue its multifaceted influenza surveillance systems, remain flexible with antiviral use policies, and revise the vaccine policies to include the population most at risk.

## Introduction

Influenza pandemic (H1N1) 2009 virus emerged in April 2009 and quickly spread worldwide within 6 weeks. In Taiwan, the first patient infected by the pandemic (H1N1) 2009 virus was identified on 20 May, 2009. This strain of influenza soon became the predominant subtype in circulation and caused 44 confirmed deaths (the mortality rate was 1.9 deaths per million population) in Taiwan during the 2009–10 influenza season. Although WHO declared the world moving into the post-pandemic period on August 10, 2010, the influenza A (H1N1) 2009 virus continued to circulate globally. Its impact, severe complications in younger age and high-risk groups, were expected to continue during the 2010–11 influenza season [1], [2].

Taiwan is situated in Eastern Asia with 23 million in population. Since 1998, free influenza vaccines have been provided to targeted groups such as the elderly, patients with catastrophic illnesses as defined by the Bureau of National Health Insurance [3], health care workers, young children aged 0.5–6 years and school-aged children. The annual vaccination coverage for seasonal influenza in the entire population was around 10–15% in recent years.

Influenza surveillance in Taiwan includes the use of sentinel physicians through the Influenza Laboratory Surveillance Network, the Real-time Outbreak and Disease Surveillance (RODS), National Health Insurance (NHI) data, and passive reporting of influenza hospitalized cases with severe complications through the National Notifiable Disease Surveillance System (NNDSS).

The Influenza Laboratory Surveillance Network is coordinated by the Taiwan Centers for Disease Control (TCDC), and comprises of 12 collaborating laboratories aimed to survey and isolate viruses causing respiratory tract infections year round [4]. More than 275 sentinel physicians in private clinics or hospitals voluntarily participated in this surveillance. The physicians were distributed in 21 of the 22 administrative areas of Taiwan. Clinic- and hospital-based physicians each contribute to approximately 50% of the specimens collected. The network has been in operation since 1999.

RODS, developed by the University of Pittsburgh (Pittsburgh, PA), was originally designed to detect bioterrorism events or emerging diseases [5]. In Taiwan, RODS has been used to monitor influenza activity through monitoring emergency room (ER) visits [6]. There are more than 160 hospitals that participate in RODS. For the surveillance of outpatient clinics, NHI database is used. Of the 23 million people living in Taiwan, >99% are enrolled in NHI [7], making NHI a comprehensive database to monitor outpatient influenza activity.

According to the Communicable Disease Control Act, physicians are required to report notifiable diseases within the allotted time frame [8]. Influenza with severe complications was made reportable to NNDSS in 2000 [9], requiring reporting within one week. Otherwise, physicians may incur fines approximately USD 3,000–15,000. However, influenza with severe complications was not widely reported until after the 2009 influenza pandemic. Prior to the pandemic, approximately 15–35 confirmed cases were reported in any given influenza season.

Through the use of all these surveillance methods, a nearly complete picture of influenza activity nationwide emerges. This study aimed to describe the nationwide influenza surveillance findings of the pandemic (2009–10) and post-pandemic (2010–11) periods in Taiwan, and to assess the impact of influenza A(H1N1) 2009 during the post-pandemic period.

## Methods

### Defining influenza seasons

Taiwan has a tropical-to-temperate spectrum of climatic zones. The annual average temperature is 22°C with the lowest temperatures ranging from 12°C to 17°C. Winters are defined as the months of December, January and February. In this study, Taiwan's influenza season was defined as July 1 to June 30 of the following year. For the 2009–10 influenza season, this corresponded to week 26, 2009–week 25, 2010; and for the study period of the 2010–11 influenza season, this corresponded to week 26, 2010–week 13, 2011. Each year, influenza activity usually begins to rise in late October, and peaks sometime during late December to early February the following year.

### Influenza Laboratory Surveillance

For the Influenza Laboratory Surveillance Network, clinical specimens obtained from nasal or throat swabs were collected by physicians and sent to TCDC collaborating laboratories for virus isolation and identification using viral culture and/or reverse transcriptase-polymerase chain reaction (RT-PCR). Methods of virus isolation have been described previously [10]. Antigenic characterization of the influenza virus was determined using the hemagglutination inhibition (HI) assays with ferret antisera [11]. Oseltamivir-resistant influenza viruses were detected using DNA sequencing and 50% inhibitory concentration (IC_50_) analysis of neuraminidase activity [12].

### Influenza-like Illness (ILI) Syndromic Surveillance

RODS collected individual patient's *International Classification of Diseases, Ninth Revision, Clinical Modification* (ICD-9-CM) diagnostic codes from patient ER visits, covering approximately 85% of all ERs in Taiwan. ICD-9-CM codes were uploaded to RODS in real time, making the data available for daily analysis. ILI cases were detected using RODS predetermined set of ICD-9-CM codes for respiratory syndrome [13].

For the NHI database, all healthcare facilities (clinics and hospitals) submit outpatient department (OPD), ER, and inpatient ICD-9-CM diagnostic codes to the Bureau of National Health Insurance (BNHI) for reimbursement. Because BNHI provided financial incentives for clinics and hospitals to submit good quality claims data electronically within one day of each patient visit, the database had been reliable for insurance claims purposes. On weekdays, aggregated data in pre-specified ICD-9-CM diagnostic code groups by age group and geographic location were sent to TCDC electronically. ILI was defined by ICD-9-CM codes 480–487.

### Reporting of Influenza Cases with Severe Complications and Deaths

For NNDSS, ILI in a patient who had any pulmonary complications that required hospitalization, neurological complications, myocarditis or pericarditis, invasive bacterial infection, or other severe conditions requiring intensive care unit admission were reported and confirmed by RT-PCR or viral culture [6], [14]. All laboratory-confirmed patients reported to NNDSS, with onset dates during July 1, 2009–March 31, 2011, who met the case definition, were included for analysis. In addition, for the 2010–11 influenza season we analyzed characteristics of confirmed patients who died by the end of week 13, 2011, and influenza was contributory as determined by a reviewing physician or recorded on the death certificate. For patients who died, medical records, where available, were reviewed.

### Antiviral Prescriptions

Antiviral medications, including oseltamivir, zanamivir, and peramivir, were provided free of charge by TCDC to 1) patients with clinical evidence of severe influenza with complications; 2) ILI patients with danger signs that signal progression to severe disease [15]; 3) ILI patients with critical illness, chronic cardiopulmonary disease, liver disease, renal disease, or diabetes mellitus; 4) ILI patients with body mass index (BMI)≥35; and 5) ILI patients in an influenza outbreak. On January 25, 2011, free antivirals were additionally provided to patients with fever for ≥48 hours and ILI patients who had been in close contact with other ILI patients at home, school, or workplace. Each prescription was registered at the Countermeasure Management Information System operated by TCDC. We compared the weekly number of antiviral prescriptions with the number of ILI visits in RODS from week 1, 2010 through week 13, 2011.

### Comparing 2009–10 and 2010–11 Influenza Seasons

We compared the age distribution of influenza outpatient visits (ICD-9-CM code 487) and influenza A(H1N1) 2009 cases with severe complications and deaths between the 2009–10 and 2010–11 influenza seasons. Chi-square tests were used to test whether the two groups of patients were similar in age distribution.

### Ethics

Data obtained for this study was for surveillance purposes; therefore, this study was not reviewed by an institutional review board.

### Statistical methods

We used Microsoft Excel (2007) to produce figures and calculate descriptive statistics such as counts and percentages. The chi-square test, median and interquartile range (IQR) were performed using SAS (SAS Institute Inc., Cary, NC, USA), Version 9.2 of the SAS System for Windows 7.

## Results

### Responses during Pandemic and Post-pandemic Phases

To mitigate the effects of pandemic (H1N1) 2009 during the pandemic period, Taiwan established the Central Epidemic Command Center (CECC) to coordinate the use of resources and implement control measures on April 28, 2009. During November 1, 2009–April 3, 2010, 22.4% of the population received at least one dose of the pandemic (H1N1) 2009 vaccine, including 29.3% of persons aged 6 months to 6 years, 71.7% of persons aged 7–18 years, and 11.3% of persons aged ≥19 years. A total of 5.67 million doses were provided [16]. In addition to providing the pandemic (H1N1) 2009 monovalent vaccine, rapid influenza diagnostic tests and antiviral prescriptions were provided through NHI from August 15, 2009 to March 31, 2010 [17].

During the post-pandemic phase, TCDC provided stockpiled antivirals free of charge to contract clinics and hospitals for patients meeting the five specified indications. During the 2010–11 influenza season, 12.3% of the population received at least one dose of seasonal influenza vaccine, including 25.9% of persons aged 6 months–6 years, 64.4% of children in grades 1–6 (aged 7–12 years), and 37.3% of persons aged ≥65 years. For the 2010–11 influenza season, because of increasing ER visits, hospitalizations, and deaths associated with influenza A(H1N1) 2009 during the first three weeks of 2011 and only ERs of major hospitals remained on duty during the week-long holiday in week 5 (the Lunar New Year), TCDC relaxed the indications for antiviral prescriptions from week 4 to week 13, 2011.

### Virologic Surveillance

During the 2009–10 influenza season, there were 14,788 specimens tested. Of these, 3,970 (26.9%) were positive for influenza viruses using viral culture and/or RT-PCR. Among the positive specimens, there were 3,165 (79.7%) influenza A(H1N1)2009, 545 (13.7%) influenza B, 216 (5.4%) influenza A(H3N2), and 10 (0.3%) seasonal influenza A(H1N1). In comparison, during the 2010–11 influenza season, influenza A(H3N2) increased. During this period, 11,813 specimens were tested. Of these, 2,767 (23.4%) were positive for influenza viruses using viral culture and/or RT-PCR. Among the positive specimens, there were 1,264 (45.7%) influenza A(H1N1)2009, 1,010 (36.5%) influenza A(H3N2), and 489 (17.7%) influenza B. No seasonal A(H1N1) were found.

**Figure 1:**
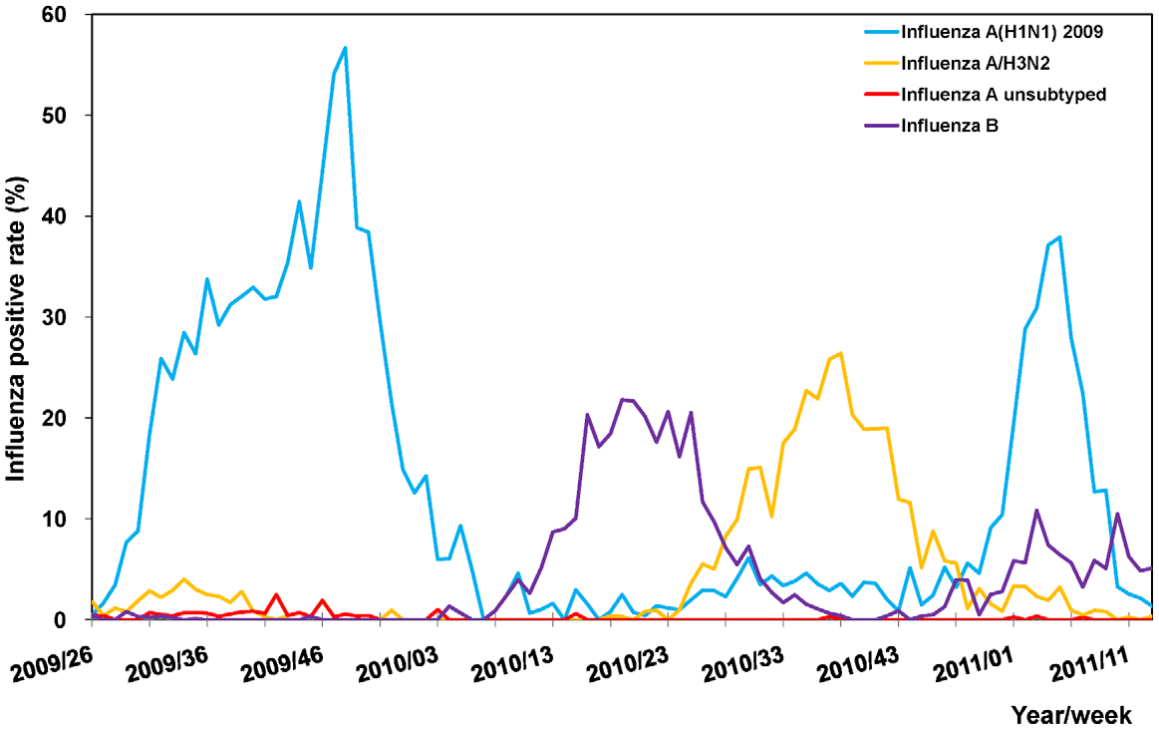
Respiratory specimen testing influenza positive rates in Taiwan, week 26, 2009–week 13, 2011.

During the 2009–10 influenza season, the activity of influenza A(H1N1) 2009 virus presented one peak in week 48 according to the influenza positive rate. Influenza activity reached nadir in week 7, 2010 (Fig. 1). Influenza A(H1N1) 2009 virus became the predominant circulating strain in week 49 of the 2010–11 influenza season. Other circulating strains during this time included influenza B and A(H3N2).

During the 2009–2011 study period, of the 146 influenza A(H1N1) 2009 viruses tested using the hemagglutination inhibition (HI) assays, 142 (97.3%) were antigenically similar to the vaccine strain, A/California/7/2009. All others showed reduced titers. Furthermore, 12 of 1,734 influenza A(H1N1) 2009 viruses tested positive for mutation H275Y in the neuraminidase gene, conferring resistance to oseltamivir. Epidemiologic investigations indicated no clustering. No transmission was found after screening of suspected cases with temporal association or in geographic proximity.

### Emergency and Outpatient Illness Surveillance

Following the first documented case of influenza A(H1N1)2009 was imported into Taiwan, patients seeking care for ILI at ERs quickly rose, peaking in week 37, 2009. OPD surveillance had its peak in week 48. This was followed by a much smaller rise during week 7, 2010, occurring in both ER and OPD surveillance because it was during the Lunar New Year, when the majority of the private clinics were closed. While these clinics were closed, patients with ILI sought help at ERs, resulting in the rise in ER surveillance. For OPD surveillance, because patients with chronic diseases stayed away during this period, the relative increase of ILI patients resulted in the small rise.

**Figure 2:**
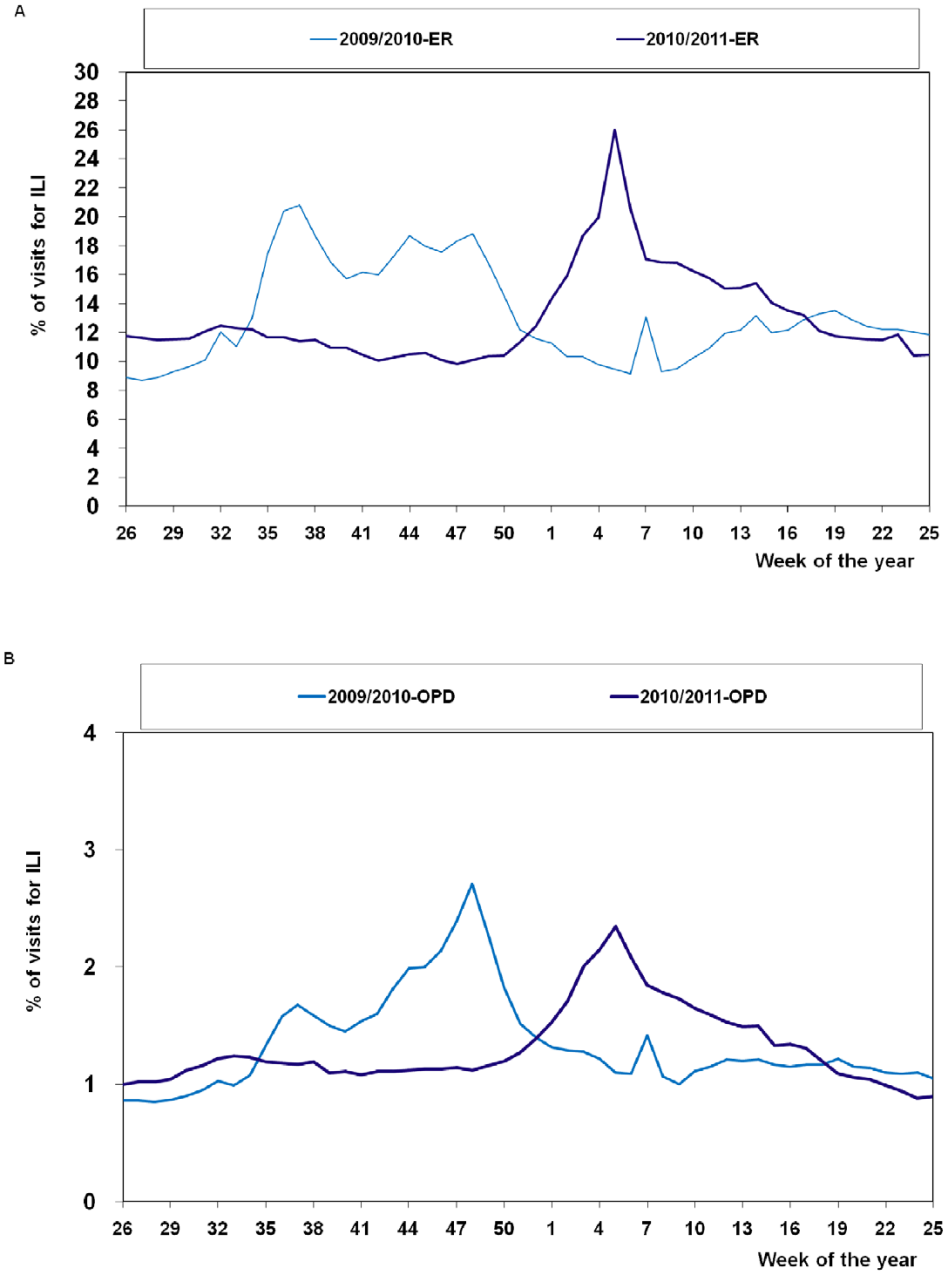
Percentages of influenza-like illness (ILI) visits in emergency room (ER) and outpatient department (OPD), week 26, 2009–week 25, 2011.

For the 2010–11 influenza season, during week 47, 2010, the percentage of ER patient-visits for ILI found through RODS increased to 9.8% and continued to increase, reaching a peak of 26.0% during week 5, 2011. The trend in OPD visits was similar. The percentage of OPD visits for ILI found through NHI increased to 1.1% during week 48, 2010 and peaked at 2.4% during week 5, 2011. This trend was similar to Taiwan's usual influenza seasons. For 2009–2010 influenza seasons, the peak percentage of ILI in ER visits was 20.8% (week 37, 2009); OPD visits was 2.7% (week 48, 2009) (Fig. 2).

### Hospitalized Cases with Severe Complications and Deaths

**Figure 3:**
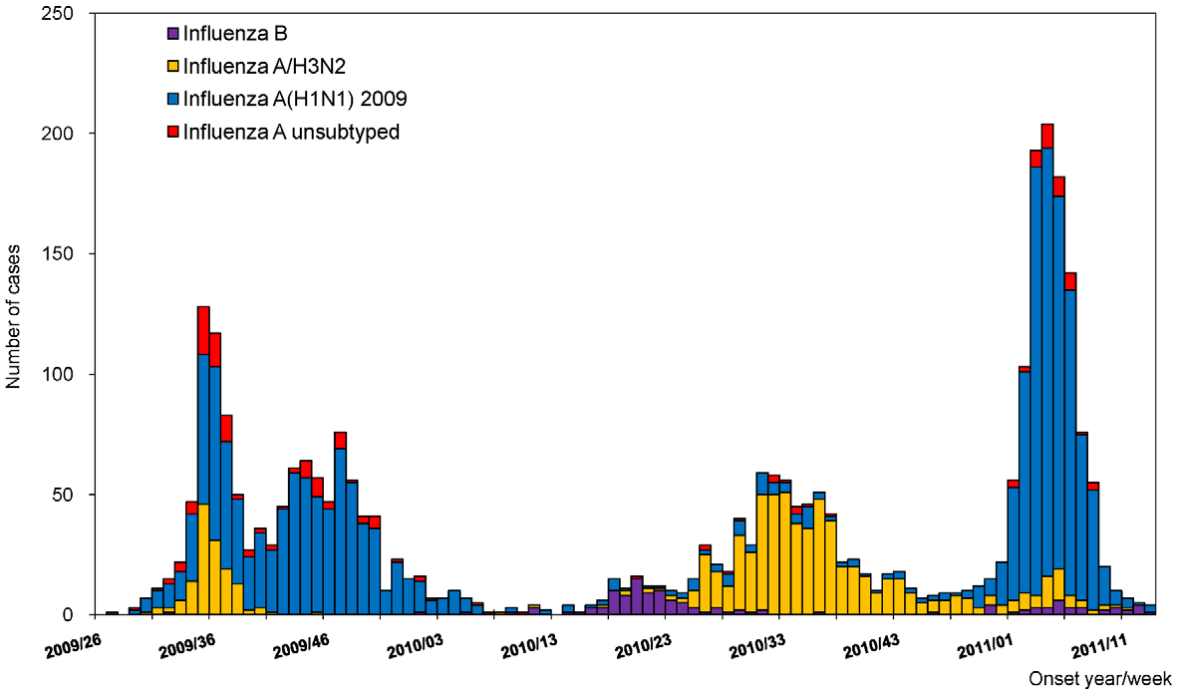
Number of influenza hospitalized cases with severe complications by week of onset in Taiwan, week 26, 2009–week 13, 2011.

During the 2009–2010 influenza season, there were 1,297 hospitalized cases with severe complications reported to the NNDSS. Among those, there were 937 (72.2%) patients infected by influenza A(H1N1) 2009 virus, 161 (12.4%) by H3N2, 111 (8.6%) by A unsubtyped, 82 (6.3%) by influenza B, and 6 (0.5%) by seasonal A(H1N1). For the 2010–11 influenza season, as of week 13, 2011, there were 1,751 hospitalized cases with severe complications reported (Fig. 3). Of the 1,751 cases reported, there were 1,040 (59.4%) patients infected by influenza A(H1N1) 2009 virus, 606 (34.6%) by H3N2, 55 (3.1%) by A unsubtyped, and 50 (2.9%) by influenza B. During weeks 26–52, 2010, influenza A(H3N2) caused disease in 548 (80.0%) of the 685 patients. In contrast, influenza A(H1N1) 2009 caused disease in 932 (87.4%) of the 1,066 patients with disease onset after January 1, 2011.

**Figure 4:**
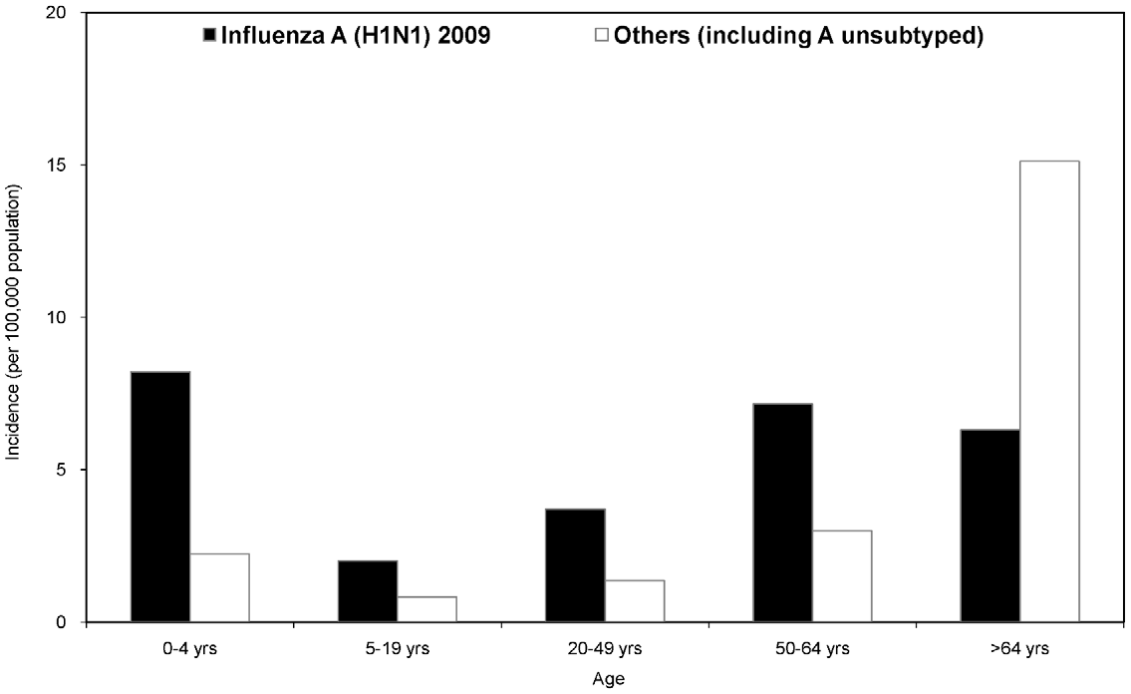
Incidence of influenza hospitalized cases with severe complications stratified by age and virus subtype in Taiwan, week 26, 2010–week 13, 2011.

The incidence of hospitalized cases with severe complications was highest among persons aged >64 years (21.4 per 100,000 population) during the 2010–11 influenza season (Fig. 4). The peak of hospitalizations occurred in week 4 of 2011. The median age of patients was 53.2 years (IQR, 31.9–70.2); 982 (56.1%) were male. There were 878 (50.1%) patients with underlying diseases, most commonly: 294 (16.8%) with metabolic diseases, 227 (13.0%) cardiovascular diseases other than hypertension, and 227 (13.0%) respiratory diseases. Admission to intensive care unit (ICU) was required for 620 (35.4%) patients.

**Figure 5:**
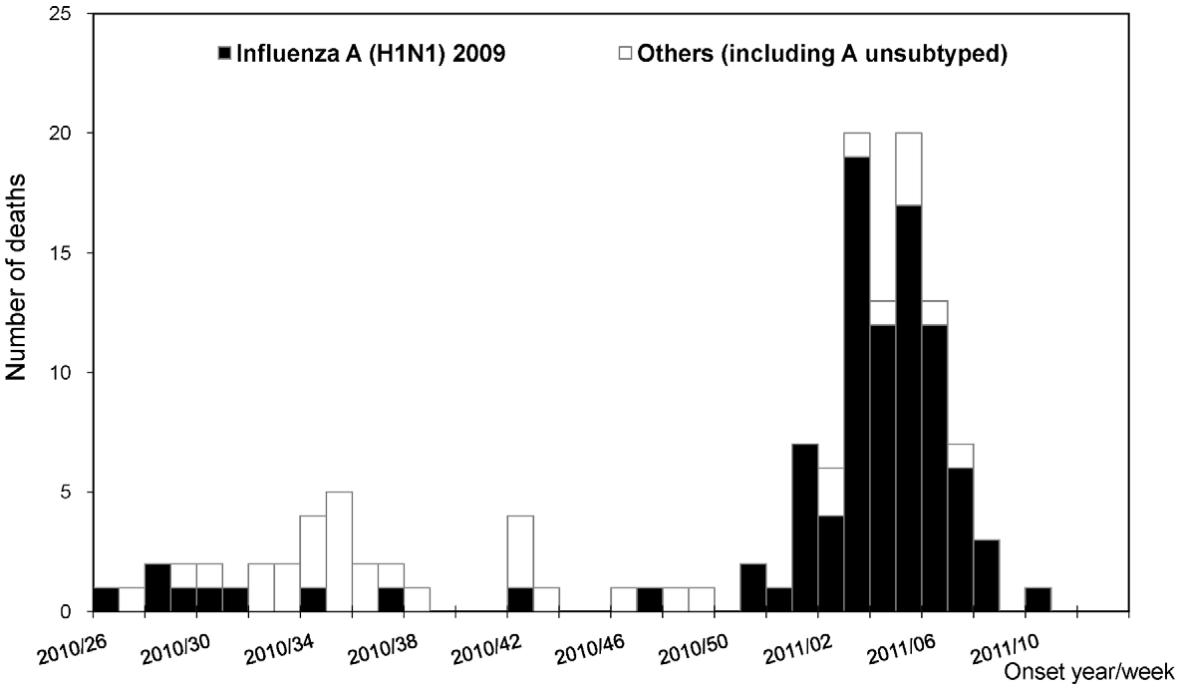
Number of influenza-associated deaths by week of onset in Taiwan, week 26, 2010–week 13, 2011.

Of the 169 (9.7%) patients who died during the 2010–11 influenza season, 128 were directly or indirectly associated with influenza (the mortality rate was 5.5 deaths per million population). By week 50, 2010, deaths were predominantly caused by H3N2 (Fig. 5). After week 51, deaths caused by influenza A(H1N1) 2009 became predominant. Deaths peaked during weeks 3–5 of 2011. Among the 128 influenza-associated deaths, 93 (72.7%) were caused by influenza A(H1N1) 2009, 30 (23.4%) by H3N2, 3 (2.3%) A unsubtyped, and 2 (1.6%) by influenza B. The median age of those who died was 56.7 years (IQR, 49.9–68.5); 89 (69.5%) were male. There were 104 (81.3%) patients who received antivirals, 41 (32.0%) within two days of symptom onset. Among those who died, 110 (85.9%) had underlying diseases. Most common underlying diseases reported were metabolic diseases (39.1%), especially diabetes mellitus (25.8%). Of the 125 patients with all dates available, on average, patients died 15 days after symptom onset (median, 12 days; IQR, 6–22 days). Among the deaths, only 2.3% of the patients received either the pandemic (H1N1) 2009 monovalent vaccine or the 2010–11 seasonal influenza vaccine.

### Antiviral prescriptions

During the post-pandemic phase, although TCDC provided free antivirals, the weekly antiviral prescriptions rarely exceeded 150 courses in 2010. After free antiviral use was relaxed in week 4, 2011, TCDC deployed 50,000 courses of antivirals to the 500 contract clinics and hospitals on January 25 (Tuesday of week 4), 2011. The numbers of antiviral prescriptions increased from 597 in week 3, to 4,055 in week 4, and to >11,000 in both weeks 5 and 6. A total of 69,866 antiviral prescriptions (oseltamivir accounted for more than 98%) were given during week 26, 2010–week 13, 2011. Of these, 61.9% were prescribed during weeks 4–8, 2011, the period when RODS showed the highest percentage of ILI patient visits to ERs.

### Comparing 2009–10 and 2010–11 Influenza Seasons

Comparison of the age distribution of influenza patients in the 2009–10 and 2010–11 influenza seasons showed that for all disease spectrum of influenza A(H1N1) 2009 infections, patients during the 2010–11 season were consistently older than the ones during the 2009–10 season (Tables 1 and 2). All three Chi-square tests for the differences in the age distribution between the two groups were statistically significant with *p*<0.0001.

**Table 1:** Comparisons of numbers of influenza A(H1N1) 2009-associated outpatient department (OPD) visits between 2009–10 and 2010–11 seasons (as of week 13, 2011)*.

Age group	2009–10 season	2010–11 season	*p* value
	n (%)	n (%)	
0–4 years	191,865 (9.2)	121,342 (7.5)	<0.0001
5–19 years	789,373 (37.9)	397,346 (24.7)	
20–49 years	653,680 (31.3)	641,696 (39.9)	
50–64 years	263,010 (12.6)	274,343 (17.0)	
≥65 years	187,399 (9.0)	175,236 (10.9)	

* 2009–10 season: from week 26, 2009 to week 25, 2010; 2010–11 season: from week 26, 2010 to week 13, 2011.

**Table 2:** Comparisons of numbers of influenza A(H1N1) 2009 cases with severe complications and deaths between 2009–10 and 2010–11 seasons (as of March 31, 2011)*.

Characteristic	2009–10 season	2010–11 season	*p* value
	n (%)	n (%)	
Severe complications			<0.0001
0–4 years	119 (12.7)	81 (7.8)	
5–19 years	370 (39.4)	87 (8.4)	
20–49 years	277 (29.5)	411 (39.5)	
50–64 years	105 (11.2)	305 (29.3)	
≥65 years	68 (7.2)	156 (15.0)	
Deaths			<0.0001
0–4 years	1 (2.3)	3 (3.2)	
5–19 years	8 (18.2)	2 (2.2)	
20–49 years	22 (50.0)	24 (25.8)	
50–64 years	9 (20.5)	49 (52.7)	
≥65 years	4 (9.1)	15 (16.1)	

* 2009–10 season: from week 26, 2009 to week 25, 2010; 2010–11 season: from week 26, 2010 to week 13, 2011.

## Discussion

Taiwan experienced influenza pandemic (H1N1) 2009 during the pandemic phase and 5.67 million doses of pandemic (H1N1) 2009 monovalent vaccine were provided from November 1, 2009 to March 31, 2010 to Taiwan's 23 million people. It is estimated that over 22.4% of the population received at least one dose of the pandemic (H1N1) 2009 vaccine in 2009 [16]. Despite the fact that the virus showed no antigenic changes during the 2010–11 season and very few drug resistance, a large epidemic of the same virus recurred during the 2010–2011 influenza season.

However, population affected by the virus shifted to those who were older. This age distribution shift in the post-pandemic period is probably the result of predominantly school-aged children being infected in 2009 and the effect of the Nationwide In-school Influenza Vaccination in 2009 [18] and the 2010–11 season. The one-dose coverage rate of the 2009 pandemic monovalent vaccine for the 3.7 million students aged 7–18 years was 71.7%. Because the most intensive influenza infections and vaccination efforts both occurred among school-aged children in 2009, this age group most likely was protected during the 2010–11 influenza season. For children aged 7–12 years, 64.4% received the 2010–11 seasonal influenza vaccines, further protecting them from influenza A(H1N1) 2009 infections. In contrast, persons aged 20–64 years and those with higher risk for influenza complications became the most vulnerable groups during the 2010–11 season. The same situation was also found in the United Kingdom, where persons aged 15–44 years and at risk groups were the major groups contributing to hospital admissions and deaths at the end of 2010, when influenza A(H1N1) 2009 was the predominant circulating strain [19].

Among persons who died in association with influenza, 85.9% had underlying diseases, mainly diabetes mellitus and cardiovascular diseases other than hypertension. However, these persons with high-risk underlying diseases were not targeted under the current vaccination program; instead, persons with the 29 groups of diseases listed as “catastrophic illnesses" in the NHI program were [3]. Because vaccination is provided free of charge, TCDC has yet to develop a good method to readily verify a person's disease status with known high-risk underlying diseases at vaccination sites. Therefore, persons in high-risk groups not marked for having a “catastrophic illness" were not included among the vaccination target groups. Countries such as the United States [20], Australia [21], and the United Kingdom [22] have targeted persons with chronic illnesses, including those with diabetes mellitus, cardiovascular diseases, and chronic respiratory tract diseases in their immunization programs. Taiwan should consider similar measures to enroll all high-risk persons into future seasonal influenza vaccination program.

During these two influenza seasons, because of the pandemic and the subsequent severe influenza seasons, TCDC changed or relaxed the indications for free antiviral use on a few occasions. It is unclear how much impact relaxing antiviral prescriptions had on mitigating the peak of influenza A(H1N1) 2009; however, having reliable real-time influenza surveillance systems routinely in use helped us to make a timely decision.

Why, then, did the large epidemic of influenza A(H1N1) 2009 with antigenicity similar to the 2009 pandemic monovalent vaccine and 2010–11 seasonal vaccine strains re-emerge in Taiwan after week 50 of 2010? We postulate that at least three factors played key roles for this phenomenon. First, the antibody against influenza A(H1N1) 2009 acquired by natural infections or vaccination may have decreased. Wang et al reported that at least 50% of the vaccinees and recovered patients lost sufficient immunity after 6 months [23]. The declining herd immunity may have resulted in the severe A(H1N1) 2009 epidemic. Second, the A/Perth/16/2009 (H3N2)-like virus was the predominant circulating virus during the first half of the 2010–11 influenza season. Although influenza A(H3N2) virus did not circulate during the typical influenza season, this virus also caused patient illness, hospitalizations, and even deaths. Starting October 1, 2010, free trivalent seasonal influenza vaccines (which include the circulating H3N2 strain) were provided to target groups. The further decline of H3N2 in November and December 2010 provided an opportunity for influenza A(H1N1) 2009 to become the predominant virus later. Third, Taiwan had a record-breaking cold winter in 2010–2011. After the first cold front reached Taiwan on December 16, 2010, the temperature remained relatively low. According to the Taiwan Central Weather Bureau, the average temperature for January 2011 was 13.7°C (56.7°F) in Taipei and 17.5°C (63.5°F) in Kaohsiung, representing the north and south, respectively, making it the coldest January since 1972 [24]. On average, the temperature in January 2011 was 2°C lower than the average of the last 30 years. Because transmission of influenza virus is highly dependent on temperature, unprecedented low temperature in a country where most households have no indoor heating might contribute to the large epidemic of influenza A(H1N1) 2009 [25], [26].

The mortality rate in Taiwan caused by influenza A (H1N1) 2009 during the post-pandemic season was higher than during the pandemic season (5.5 vs. 1.9 deaths per million population, *p*<0.0001). These two numbers were similar to the death risks of influenza A (H1N1) 2009 in England during the second and the first waves of pandemic in the 2009–10 season (5.4 vs. 1.6 deaths per million population), respectively [27]. Both Taiwan and England reported higher mortality rate during the second wave compared to the first wave. Comparable or even more severe post-pandemic period was observed in the same winter in the several European countries [28], such as UK [19], [29], [30], Greece [31], and Spain [32]. Multiple waves of pandemic influenza were also reported in past pandemics [33].

The findings in this study had some limitations. First, the number of influenza-associated hospitalizations and deaths may be underdiagnosed or underreported because data were derived from a passive surveillance system. Second, using chart review and death certificate to determine whether influenza was contributory to death was subject to misclassification bias. Third, to track ILI activity, we used ICD-9-CM administrative data for syndromic surveillance, which may have lower specificity [34] and coding errors [35].

In conclusion, reemergence of influenza A(H1N1) 2009 during Taiwan's 2010–2011 influenza season had an intense activity with age distribution shift, that during the 2010–11 influenza season, influenza A(H1N1) 2009 patients of all severities were consistently older than those during the 2009–10 season. Most patients with severe disease or died did not receive the current season's influenza vaccine. To further mitigate the impact of future influenza epidemics, Taiwan must continue its multifaceted influenza surveillance systems, remain flexible with antiviral use policies, and revise the vaccine policies to include the population most at risk.
